# Supplementary material for: Transcriptome Analysis Reveals Vimentin-Induced Disruption of Cell–Cell Associations Augments Breast Cancer Cell Migration
Source: Cells. 2022 Dec 13;11(24):4035. doi: 10.3390/cells11244035 (PMC9776984; doi:10.3390/cells11244035)
Supplement: Supplementary file 1 [file cells-11-04035-s001.zip › DEGs and enrichment analysis.zip!/DEGs and enrichment analysis/WT_VIMvsVector GO enrichment/UP/WT_VIMvsVector_up.CCbar.pdf]

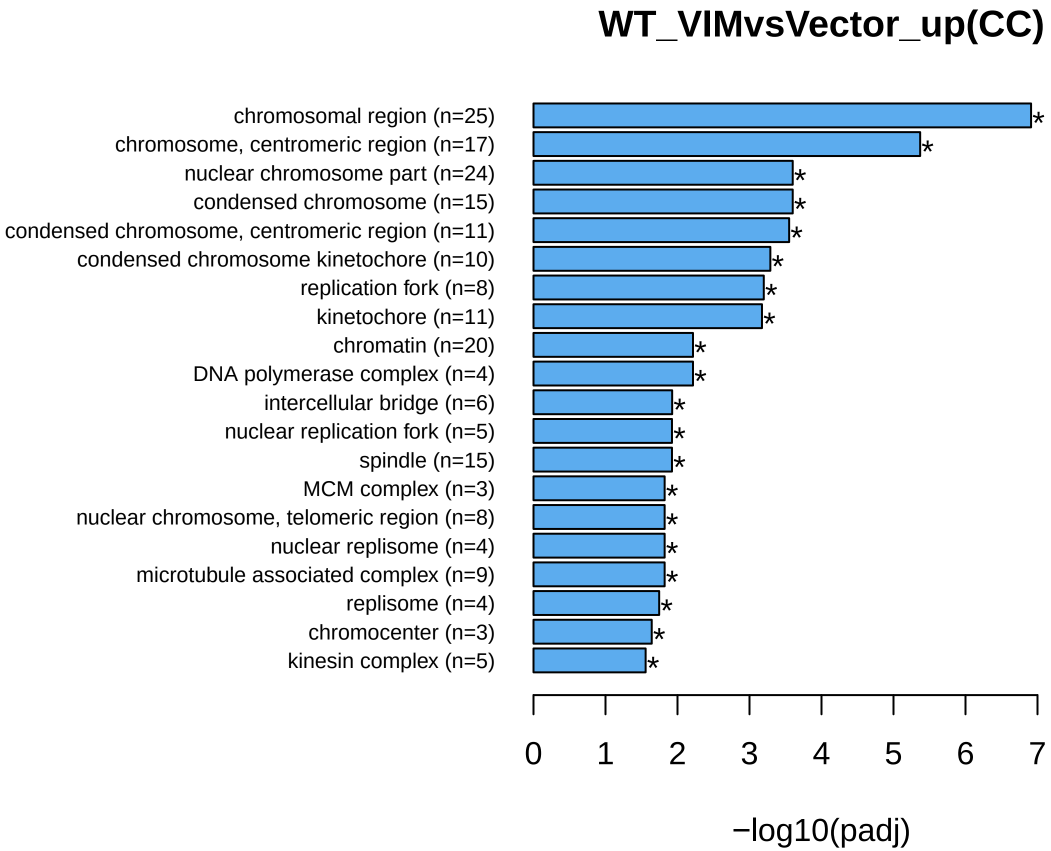
WT_VIMvsVector_up(CC)
chromosomal region (n=25)
chromosome, centromeric region (n=17)
nuclear chromosome part (n=24)
condensed chromosome (n=15)
condensed chromosome, centromeric region (n=11)
condensed chromosome kinetochore (n=10)
replication fork (n=8)
kinetochore (n=11)
chromatin (n=20)
DNA polymerase complex (n=4)
intercellular bridge (n=6)
nuclear replication fork (n=5)
spindle (n=15)
MCM complex (n=3)
nuclear chromosome, telomeric region (n=8)
nuclear replisome (n=4)
microtubule associated complex (n=9)
replisome (n=4)
chromocenter (n=3)
kinesin complex (n=5)
0
1
2
3
4
5
6
7
−log10(padj)
